# FINANCIAL CAPABILITY AMONG OLDER ADULT IMMIGRANTS, A SYSTEMATIC REVIEW

**DOI:** 10.1093/geroni/igad104.3357

**Published:** 2023-12-21

**Authors:** Bruna Lopez, Barbara Mendez Campos, Erika Sabbath

**Affiliations:** Boston College, Boston, Massachusetts, United States; Boston College, Boston, Massachusetts, United States; Boston College, Boston, Massachusetts, United States

## Abstract

The Financial Capability approach assesses individuals’ financial knowledge and behaviors within the context of opportunities in the environment. Scholars and practitioners have documented how immigration status can influence individuals’ access to and eligibility for financial products and services, due to federal, state, and community rules and norms. Those who lack immigration status (i.e., undocumented immigrants) often face additional financial hurdles. A separate line of research has shown how financial capability can influence retirement savings, yet this did not focus on immigration status. As such, this systematic review seeks to understand how variations in legal immigration status impact the financial capability and long-term financial wellbeing of older adult immigrants in the United States. The current review utilized standardized procedures and protocols to identify articles related to financial capability, immigration status, and retirement savings, resulting in an initial list of 3,599 articles. Three studies published before May 2023 were included in the final narrative synthesis due to their inclusion of immigrants over 55. Results demonstrate that financial capability studies of older adult immigrants that met eligibility criteria have centralized Asian individuals, particularly Chinese, Taiwanese, and Korean populations. Furthermore, studies indicate that increasing financial capability among older immigrants can strengthen their financial security, yet these studies focus less on the impact of immigration status. Programs, services, or products operating on the financial capability paradigm warrant continued research as older immigrants have unique attributes (e.g., language, culture) and require careful design, implementation, and evaluation of efforts seeking to target long-term financial wellbeing.

